# Association between dietary patterns and metabolic syndrome and its components in quilombola women: A population-based cross-sectional study in Alagoas, Northeast Brazil

**DOI:** 10.1186/s40795-026-01402-4

**Published:** 2026-06-19

**Authors:** Lídia Bezerra Barbosa, Nancy Borges Rodrigues Vasconcelos, Ewerton Amorim dos Santos, Tamara Rodrigues dos Santos, Thays Ataide-Silva, Haroldo da Silva Ferreira

**Affiliations:** 1 Faculty of Nutrition, Federal University of Alagoas, Campus A. C. Simões, BR 104, km 14, Tabuleiro dos Martins, Maceió, Alagoas Brazil; 2 Federal University of Alagoas, Maceió, Alagoas Brazil; 3 Faculty of Medicine, Federal University of Alagoas, Maceió, Alagoas Brazil

**Keywords:** Metabolic Syndrome, Food Consumption, Western Diet, Group with African Ancestry, Healthy Diet

## Abstract

**Background:**

Investigation of dietary patterns provides an integrative model for examining the role of diet in chronic diseases. In this context, this study evaluated dietary patterns and their association with metabolic syndrome (MetS) and its components among quilombola women.

**Methods:**

A population-based cross-sectional study was conducted with women aged 19 to 59 years living in quilombola communities in Alagoas, Brazil. Socioeconomic, demographic, anthropometric, clinical, and dietary intake data were collected using a 24-hour dietary recall. Metabolic syndrome was defined according to the Joint Interim Statement criteria (abdominal obesity, hypertriglyceridemia, low HDL cholesterol, hypertension, and hyperglycemia assessed by HbA1c instead of fasting plasma glucose). Dietary patterns were identified by factor analysis and classified into quartiles. Associations were estimated using prevalence ratios obtained by Poisson regression with robust variance.

**Results:**

The sample included 895 women (mean age: 38.9 ± 11.0 years), of whom 48.3% had MetS. Seven dietary patterns were identified, none of which were associated with MetS. Moderate adherence to the “meat and beans” pattern was associated with a lower prevalence of hypertriglyceridemia (PR = 0.76; 95% CI: 0.58–0.99). The “dairy and soups” pattern was associated with a lower prevalence of hyperglycemia (PR = 0.68; 95% CI: 0.49–0.93) and low HDL cholesterol (PR = 0.89; 95% CI: 0.80–0.99). Higher adherence to the “cereals/roots, oils, and infusions” pattern (PR = 1.10; 95% CI: 1.01–1.19) and higher adherence to the “fruits” pattern in the second quartile (Q2) (PR = 1.10; 95% CI: 1.02–1.20) were associated with abdominal obesity.

**Conclusion:**

No dietary pattern was directly associated with metabolic syndrome; however, relevant associations were observed with specific metabolic components - particularly hypertriglyceridemia, hyperglycemia, low HDL cholesterol, and abdominal obesity -, highlighting the importance of dietary profiles in the metabolic health of quilombola women.

**Supplementary Information:**

The online version contains supplementary material available at https://doi.org/10.1186/s40795-026-01402-4.

## Background

 Metabolic syndrome (MetS) comprises a group of interconnected metabolic abnormalities and is associated with an elevated risk of diabetes, cardiovascular disease, and mortality [1, 2]. Considering the Brazilian population, a prevalence of approximately 38.4% was observed, with females (41.8%) being more affected than males (34.6%) [3]. In addition to gender, ethnicity also influences the occurrence of MetS [4]. In Brazil, among the Afro-descendant population living in quilombola communities, prevalences ranging from 25.8% [4] to 55.4% [5] have been reported. Therefore, the prevalence of MetS varies across different epidemiological settings in which studies are conducted, even within the same country. While some of these differences can be attributed to differences in methodological criteria, factors such as gender, age, race, and ethnicity also play an important role in these discrepancies [3].

Quilombola communities are human settlements formed by descendants of slaves who were trafficked to Brazil between the 16th and 19th centuries. They are characterized by low socioeconomic status and high social vulnerability [6, 7]. In this scenario, there is a pattern of variables that together form the social determinants of health: low income, limited education, precarious housing conditions, reduced access to healthcare services, and food insecurity. It is well-documented that these conditions predispose individuals to the development of various diseases, including non-communicable chronic diseases (NCDs), such as MetS [7, 8].

Research across different populations indicates that a healthy dietary lifestyle is essential for preventing the development of MetS [9–11]. However, it is known that dietary choices are complex and involve factors beyond the nutrients present in the diet [12–14]. Therefore, given that each food is not consumed in isolation and that the components of the diet interact, it is challenging to detect associations between the intake of these constituents and chronic diseases, particularly MetS and its components [12, 13].

An appropriate approach involves studying dietary patterns. Analyzing dietary patterns allows evaluation of the effects of multiple food groups on promoting or deteriorating the population’s health [12, 13, 15, 16].

It is important to understand the dietary patterns of socially vulnerable populations, such as quilombola communities, where high prevalences of MetS and its components are observed, in order to establish relationships between these conditions and facilitate the development of appropriate interventions. Despite this, in Brazil, no studies have analyzed dietary patterns among quilombolas and their association with MetS, particularly among women.

Thus, the aim of this study was to investigate the dietary patterns of quilombola women in Alagoas and their association with MetS and its components.

## Methods

### Study design, study location, ethical aspects, and sampling plan

This is a cross-sectional household survey study, part of a broader project titled ‘Health Diagnosis and Food and Nutritional Security of families in the remaining quilombo communities in the state of Alagoas, Northeast Brazil’ (Quilombola Project). This study was conducted in accordance with the ethical principles of the Declaration of Helsinki and was approved by the Research Ethics Committee of the Federal University of Alagoas. (CAAE 33527214.9.0000.5013).

For the Quilombola Project sampling plan, an umbrella study with multiple subprojects, the family was the unit of analysis. Due to the diversity of objectives proposed for the survey, a50% prevalence was assumed for all outcomes of interest, ensuring the maximum possible sample size. Thus, considering a 95% confidence interval and the estimated number of 6465 households in quilombola communities in Alagoas, as reported in official data available at the time of the study, the required sample size was calculated using the StatCalc module of Epi Info software [17, 18].

To obtain the planned sample and ensure broad territorial coverage of quilombola communities in the state, it was established that data collection would be conducted in approximately 50% of the certified communities. The list of communities certified by the Palmares Cultural Foundation served as the sampling frame. Using systematic sampling, 34 of the 68 existing communities were selected from this list [17, 19]. Communities were considered the primary sampling units, and eligible women were identified within households in the selected communities.

Because the number of households varied across communities, the proportion of selected communities did not exactly to match the proportion of families in the state. However, this strategy allowed the inclusion of communities distributed across multiple municipalities and regions of Alagoas, ensuring adequate geographic representation.

For the present study, all women aged 19 to 59 years, residing in the selected communities and with information available on the components of MetS were eligible. After applying the exclusion criteria (explained below), a sample of 895 women was obtained. With this sample size, the sampling error was calculated a posteriori, using the prevalence of MetS found in this survey (48.3%) and the estimated universe of women in the target age group. To estimate the population size (the universe to be represented), the total number of quilombola families residing in Alagoas in 2015 (*n* = 6465) [20] was considered, assuming an average of one woman per family. In addition to this prevalence and universe, the calculation, performed in the StalCalc module of Epi Info 7.2 software (CDC, Atlanta, USA), also considered a design effect correction factor of 1.2 and 68 clusters. For a 95% confidence interval (95% CI), a sample of 895 women yields an estimated sampling error of about 3.4%.

A post hoc power analysis was performed using Epi Info software, considering the primary association investigated in this study. Based on the prevalence of metabolic syndrome observed among exposed and non-exposed groups (53.1% and 43.0%, respectively), and assuming a 95% confidence level and 80% statistical power, the estimated sample size required to detect an association of this magnitude ranged from 775 to 815 participants. As the analytical sample included 895 women, the study was considered adequately powered to detect associations of similar magnitude.

In studies identifying dietary patterns, the sample size should ideally be at least 100 and at least five times the number of variables analyzed [21]. The ratio of individuals to food groups in the present study met this assumption.

### Eligibility and exclusion criteria

Eligible women were those with available dietary intake data and the information required to define MetS. Women who were pregnant or lactating, who reported alcohol consumption on the day of the biochemical examination, or who reported implausible energy intake (< 400 or > 4800 kcal/day) were excluded from the analysis based on cut-off points previously used in studies assessing dietary intake [19, 22].

### Data collection

Data collection took place at a single time point (home visit) between April 2017 and January 2018. Structured forms [23] that had been pre-tested in a pilot study were used [19]. All interviewers received training to standardize procedures and minimize errors in the form application. The collected information included socioeconomic characteristics, lifestyle, health, and dietary intake. Blood pressure was measured during the interviews, and anthropometric data were collected. Biochemical tests were conducted at a predetermined location in each community, to which the women were referred [19].

The economic classification was established according to the criteria of the Brazilian Association of Research Companies [24] (Associação Brasileira de Empresas de Pesquisa), classifying families into economic classes A, B1, B2, C1, C2, and D + E.

Blood pressure was measured in duplicate, with the individual seated and after 15 min of rest, using digital devices from the Omron^®^ brand, model HEM-7200. If there was a difference greater than 20 mmHg between the two measurements, a third measurement was taken. A minimum interval of five minutes was observed between measurements. For analysis, the most discrepant measurement was discarded, and the average of the valid measurements was calculated [17, 19].

The anthropometric variables assessed included Body Mass Index (BMI), neck circumference (NC), waist circumference (WC), and waist-to-height ratio (WHtR). BMI was calculated from weight and height data. All measurements were taken with the women standing and wearing lightweight clothing. Weight was measured using a digital scale from Seca^®^, model 813, with a capacity of up to 200 kg and sensitivity of 100 g; height was obtained using a portable stadiometer from Seca^®^, model 213, with a capacity for measurements up to 205 cm. WC and NC were measured using a non-stretchable measuring tape with a sensitivity of 0.1 cm and a capacity of 150 cm. The CC was measured at the midpoint between the iliac crest and the lowest rib’s outer edge. CP was assessed at the midpoint of neck height, at the level of the cricothyroid cartilage. WHtR was calculated by dividing WC by height.

Biochemical tests (glycosylated hemoglobin - HbA1C, triglycerides - TG, and high-density lipoprotein - HDL) were performed without prior fasting [25, 26]. Conducting biochemical tests without fasting is in accordance with the position of the Updated Brazilian Guideline on Dyslipidemia and Atherosclerosis Prevention, which states that fasting is not necessary [25]. Laboratories are instructed to report in the results the different sample collections: without fasting and with 12-hour fasting. Additionally, the use of HbA1C is not included in the Brazilian Guideline on Dyslipidemia and Atherosclerosis Prevention, based on the recommendation of the Brazilian Diabetes Society, which indicates that its measurement is independent of the individual’s fasting status [26]. Biochemical tests were performed on blood drops obtained by puncturing the fingertip using disposable lancets. The HbA1C level was measured using the Alere NycoCard Reader II^®^ device (Abbott, USA), while the HDL and TG measurements were carried out using the Alere Cholestech LDX System^®^.

Unlike all other variables, biochemical tests were obtained only from a subsample of eligible women, with a ratio of one in every three women. This allocation was performed randomly and systematically, starting with the first woman selected on that data collection day.

### Variables

#### Outcome

MetS was defined according to the harmonized criteria of the Joint Interim Statement (JIS) [27] with adaptation [19]: (a) Abdominal obesity: WC ≥ 80 cm; (b) Hypertriglyceridemia: TG ≥ 175 mg/dL or undergoing drug treatment for elevated TG; (c) Low HDL: HDL < 50 mg/dL or undergoing drug treatment for low HDL; (d) Hypertension: systolic blood pressure ≥ 130 and/or diastolic blood pressure ≥ 85 mmHg or undergoing drug treatment for hypertension; (e) Hyperglycemia (diabetes): HbA1C ≥ 6.5% or undergoing drug treatment for diabetes. The adaptation consisted of replacing fasting glucose, used by the JIS, with HbA1C, which is also recommended by the Brazilian Diabetes Society [26]. The MetS was characterized by the presence of at least three criteria from the JIS. Women with two or fewer positive criteria for MetS were classified as not having MetS.

#### Exposure variable

The exposure variable was food consumption, represented by dietary patterns. Dietary intake was obtained through the application of a single 24-hour dietary recall (24hDR). To reduce memory bias and facilitate the quantification of food portions, a photographic food record album was employed [28].

The amounts of food consumed, reported in household measures, were converted into grams and milliliters using household measurement Tables [28–31]. For the analysis of the nutritional composition of foods, the U.S. Department of Agriculture’s Food Composition Table was used as a reference [32]. In the case of foods that are part of the Brazilian dietary culture, national references were used [33–36].

### Definition of food groups

All food items mentioned in the 24hDR were listed and grouped according to similarities in their nutritional composition, preparation method, main ingredient, culinary use, and consumption patterns within the study population. Subsequently, the consumed food groups were identified for use in identifying dietary patterns. These groups were organized according to the food group classifications already defined in the Food Guide for the Brazilian Population [37], which is structured by culinary use and nutritional function rather than by level of food processing, followed by the groups listed in the Brazilian Household Budget Survey (Family Budget Survey) 2008–2009 [38], in that order. This approach is commonly used in dietary pattern analyses to reduce the dimensionality of dietary data and facilitate the interpretation of dietary patterns.

Each item within the identified food groups was expressed in grams (g) or milliliters (mL) per day. When a particular food was not consumed by the woman, its value was recorded as 0 [39]. For beverages (fruit juices, coffee, and teas). added sugar was calculated separately. As for seasonings and spices, these were separated from recipes, and also calculated separately, considering that in 80% of the 24hDR they were mentioned in the preparation recipes and were often used around 2 to 3 times a day.

All food items were maintained due to the global analysis of the food group considered; to exclude a food group, the adopted criterion would be that it had been mentioned by less than 3% of the interviewees [39].

From the defined food groups, dietary patterns were derived *a posteriori*. Each dietary pattern obtained was given a qualitative designation based on the characteristics of the food groups retained in each pattern.

### Covariates

- Demographic and Socioeconomic Variables: age group (19–29, 30–39, 40–49 and 50–59); self-reported skin color (black/brown or other); education level (never studied, 1–4 years, 5–7 and ≥ 8 years), marital status (single, married or widowed/divorced); economic class (A + B+C or D + E); participation in government social programs (yes or no); family income (≤ 1 minimum wage and > 1 minimum wage); employment status (employed or unemployed); food insecurity according to the Brazilian Food Insecurity Scale - EBIA [40] (yes or no).
- Variables related to lifestyle and recent clinical conditions: included alcoholism (yes or no); smoking (yes or no); physical activity level (PAL), obtained by applying the International Physical Activity Questionnaire (IPAQ), short version [41]; and, presence of self-reported health problems in the last 15 days was recorded (yes or no)
- Anthropometric variables: Overweight, defined as BMI ≥ 25 kg/m² [42] (yes or no); elevated NC, defined as ≥ 34 cm (yes or no) [43]; WHtR: adequate (< 0.53) or elevated (≥ 0.53) [44].

### Statistical analysis

The data, except for dietary consumption, were independently double-entered into the Epi Info software version 3.5.3. Dietary consumption data were entered only once into the Dietpro^®^ Clinico software version 6.0; a verification procedure was conducted by comparing the electronic records with the original data collection forms. Data consistency checks were conducted to identify possible inconsistencies, implausible values, and data entry errors. When discrepancies were identified (e.g., typing errors or values outside the expected range), the original data collection forms were consulted, and corrections were made when necessary [19]. In cases of disagreement between entries, the value recorded in the original data collection form was considered the reference for correction.

Descriptive analyses were conducted for all variables. The results were expressed in absolute and relative frequencies for categorical variables. Analyses were conducted using available data for each variable; therefore, denominators may vary due to missing values. No imputation procedures were applied, and analyses were based on complete cases for the variables included in each analysis.

Dietary patterns were identified by exploratory factor analysis using principal component analysis (PCA), followed by orthogonal Varimax rotation. Bartlett’s sphericity test (*p* ≤ 0.05) and the Kaiser–Meyer–Olkin (KMO) measure of sampling adequacy (≥ 0.50) were used as assumptions for performing the factor analysis [45]. The determination of the number of factors was based on Kaiser’s criterion, selecting components with eigenvalues greater than 1.0, and on visual inspection of the scree plot [45]. Factor retention also considered the interpretability of the factor structure and the nutritional coherence of the food group loadings. The derivation of dietary patterns followed an exploratory a posteriori approach. Factors were rotated using orthogonal Varimax rotation. Food groups with factor loadings ≥ 0.30 or ≤ − 0.30 were considered to contribute to the pattern, and dietary patterns were named according to the food groups with the highest factor loadings, reflecting the predominant foods characterizing each pattern [46, 47].

We performed sensitivity analyses to assess the robustness of the retained dietary patterns. Alternative factorial solutions with fewer retained components were explored using parallel analysis (Horn’s method) and different rotation methods (Varimax and Promax rotations). The interpretability and nutritional coherence of the retained factors were compared with each other.

An individual score was obtained for each factor, and these values were categorized into quartiles (Q1 to Q4). Q1, representing the lowest adherence, was taken as the reference for each dietary pattern. Quartile 2 (Q2) was considered low adherence, quartile 3 (Q3) moderate adherence, and quartile 4 (Q4) was considered higher adherence. Analyses were conducted by comparing quartiles Q2, Q3, and Q4 with Q1.

The primary outcome (MetS) was compared with different covariates to observe statistical differences between categories. For this purpose, the *Pearson χ*² test was used; crude and adjusted prevalence ratio (PR) with respective 95% confidence intervals (95% CI) were established using Poisson regression with robust variance adjustment, including in the multiple analysis the covariates that in the crude model showed a significance level of up to 20% (*p* ≤ 0.2). Additionally, before the aforementioned analysis, to avoid the problem of multicollinearity, variables that exhibited high correlations with each other (*r* = 0.7) were identified. According to the Pearson correlation matrix, only the anthropometric variables BMI and WHtR (*r* = 0.73) showed this characteristic. Therefore, only BMI was retained in the multivariable analysis.

The multiple models were organized into 3 blocks [19]: Model 1 (adjusted for demographic and socioeconomic characteristics); Model 2 (variables from Model 1 with a significance level of 5% plus information related to lifestyle and health); and Model 3 (variables from Model 1 and 2 with a p-value < 0.05 plus anthropometric data). At each of the three levels of analysis, there was a successive elimination of non-significant variables (backward stepwise). The final adjusted model consisted of all variables that remained in Model 3. The statistical analysis was conducted using Stata/SE version 12.1 software (StataCorp LP. College Station, TX, USA).

## Results

### Food groupings used in the dietary pattern analysis

The foods in the 24hDR were grouped into 14 food groups (Table 1) according to their composition, preparation method, and main ingredient. The proportion of variance explained by each factor is presented in Table 2.

**Table 1:** Distribution of food by food groups

Food groups	Description of foods or preparations
Sugar and candies	Cake with or without filling, sweet pie of any flavor, stuffed biscuit, sweet of any kind, ice cream/popsicle chocolate powder, breakfast cereal, sugars (brown, crystal, demerara), sweetener, honey, confectionery, peppermint candy, lollipop, chewing gum, cocada (sweet coconut candy), peanut candy, churros, condensed milk, ‘pé-de-moleque’, pudding of any flavor, tortilette, mousse, shakes, regular/light/diet/zero soda, sweetener, reconstituted juices/powdered soft drinks, milk/chocolate drink (liquid)
Alcoholic beverage	Beer, cognac, cachaça, wine, etc.
Coffee and teas	Coffee and teas (chamomile, lemon balm, fennel, lemongrass,…)
Broths and soups	Soup (meat, vegetable), chicken soup, meat broth, fish broth
Meat and eggs	Beef (cooked, roasted, ground, smoked, grilled, fried), shank, steak, pork loin, liver, gizzard, beef stroganoff, chicken (grilled, roasted, boiled, fried), fish (roasted, fried, cooked), egg (boiled, omelette), processed meats (mortadella, ham, turkey breast, sausage, sausage, hamburger)
Cereals, breads, pasta, roots, tubers and derivatives	Rice (polished, parboiled, brown, etc.), rice flour, sweet rice, oats, oat flour/flaked oats, barley, corn-based preparations (roasted corn, boiled corn, canned green corn, couscous, cornmeal corn, hominy, mungunzá) sweet or salty popcorn (homemade/buttery), wheat flour, breads in general (French, Creole, silk, wallet, sweet, wholemeal, shaped, etc.), simple cake, simple cake corn/carrot, cassava, pasta with or without sauce, lasagna, macaroni, instant noodles, pancake, donut biscuit, cornstarch biscuit, cream cracker biscuit, sweet biscuit without filling, water and salt biscuit, seven layer biscuit, wholemeal biscuit, Yam, sweet potato, English potato, cassava, English mashed potato, French fries, tapioca, ‘farofa’ (fried manioc flour), ‘beiju’ (a type of Brazilian tapioca), cassava flour
seasonings and sauces	Seasoning (cumin), paprika, salt, vinegar, industrialized tomato sauce, ketchup, pepper, industrialized seasonings, cinnamon, cloves, bay leaves, Worcestershire sauce.
Fruits	Banana, orange, pineapple, guava, mango, grape, apple, pear, lemon, genipap, acerola, papaya,… Natural fruit juices, coconut water, coconut
Snacks/ fast foods	Pizzas, pastries (fried or baked), coxinha, savory pie of any flavor, industrialized snacks (cheese flavor, ham, etc.), croissant, pie,…
Vegetables	Pumpkin, gherkin, carrot, chayote, okra, tomato, cucumber, onion, pepper, gherkin, beetroot, garlic,,,,
Beans	Various types of beans, broad beans, soy/textured soy protein, canned peas or fresh grains, peanuts, bean-based preparation (‘baião-de-dois’- rice and black-eyed peas cooked together)
Dairies	Milk (whole/skim), cheeses, milk cream, cream cheese, and fruit shakes made with milk
Oils and fats	Soybean oil, butter, margarine, olive oil, lard, mayonnaise, pork bacon
Greens	Lettuce, cabbage, kale, coriander, chives, mint, orégano…

**Table 2:** Factor loadings and dietary patterns in Quilombola women, Alagoas, Brazil, 2018

Food groups	Factors						
	Factor 1	Factor 2	Factor 3	Factor 4	Factor 5	Factor 6	Factor 7
	Meat and beans pattern	Cereals/roots, oils, and infusions pattern	Vegetables and seasonings pattern	Sugars and fast food pattern	Alcohol and greens pattern	Dairy and soups pattern	Fruits pattern
Meats and eggs	0.7236*	0.1151	0.1669	0.0008	-0.0370	-0.1097	-0.0089
Beans	0.6048*	-0.1199	-0.0077	-0.2129	0.1071	0.0855	0.0983
Cereals, breads, pasta, roots, tubers and derivatives	0.4367	0.5384*	0.0914	0.0970	-0.0819	0.3401	-0.0181
Oils and fats	-0.2351	0.6952*	0.2035	-0.0313	-0.1610	-0.1256	-0.0365
Coffee and teas	0.1458	0.6945*	-0.2184	-0.0068	0.2526	-0.0398	0.0704
Condiments/ seasonings and sauces	0.2933	0.1060	0.5785*	0.2448	-0.0016	0.1066	-0.2210
Vegetables	0.0608	0.0011	0.7281*	-0.0556	-0.0870	-0.0469	0.1207
Sugar and candies	-0.1213	0.1155	0.1080	0.6909*	0.0830	0.0898	-0.0174
Snacks	-0.0243	-0.1124	-0.0484	0.7373*	-0.0454	-0.0401	0.1210
Alcoholic beverage	0.0670	0.0592	-0.1049	0.0255	0.8390*	-0.0199	-0.0598
Greens	-0.0750	-0.0956	0.5344*	0.0153	0.5398*	-0.0329	0.0776
Dairy	0.0588	-0.0993	-0.0478	0.0946	-0.0598	0.8197*	-0.1275
Broths and soups	0.2535	-0.1409	-0.1008	0.2002	-0.1057	-0.4931*	-0.4551
Fruits	0.1559	-0.0111	0.0251	0.1681	-0.0582	-0.1132	0.8368*
**Variance explained (%)**	**9.74**	**9.73**	**9.38**	**8.70**	**8.14**	**7.89**	**7.34**
**Eigenvalue**	**1.36**	**1.36**	**1.31**	**1.21**	**1.13**	**1.10**	**1.03**

* Higher factor loading of the food group

### Factor-loading matrix for the identified dietary patterns

**Fig. 1:**
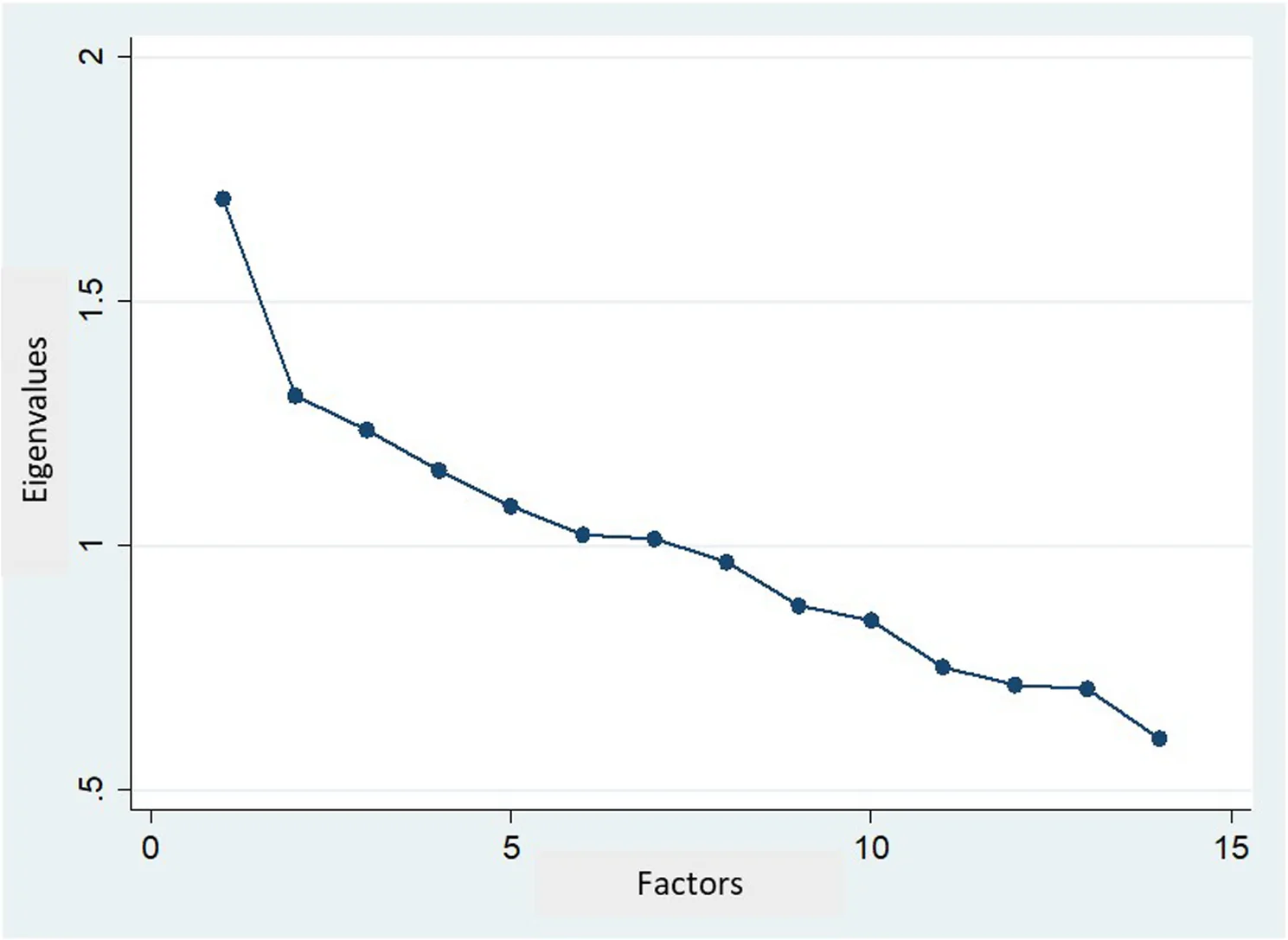
Scree plot of eigenvalues from factor analysis using 24-hour dietary recall among quilombola women. Legend: Eigenvalues are plotted against the number of extracted factors to support the selection of dietary patterns

Factor analysis identified seven dietary patterns (Fig. 1), which together explained 60.9% of the total variance in food intake (Table 2).

Sensitivity analyses using parallel analysis suggested a solution with four retained factors (Supplementary Table 1). However, this approach resulted in broader dietary patterns with less nutritional and cultural interpretability due to the merging of food groups representing distinct dietary behaviors in the studied population. Therefore, the seven-factor solution was retained as it better represents the diversity of dietary practices observed among quilombola women.

The factor-loading matrix for the identified dietary patterns is presented in Table 2. The dietary patterns were named: Factor 1, or meat and beans pattern; Factor 2, or cereals/roots, oils, and infusions pattern; Factor 3, or vegetables and seasonings pattern; Factor 4, or sugars and fast food pattern; Factor 5, or alcohol and greens pattern; Factor 6, or dairy and soups pattern; and Factor 7, or fruits pattern (Table 2).

The “meat and beans” pattern was characterized by high consumption of red meat, poultry, fish, eggs, and various types of beans. The “cereals/roots, oils, and infusions” pattern showed higher consumption of foods such as rice, cassava flour, corn, homemade popcorn, couscous, yam, sweet potato, tapioca, beiju (a traditional Brazilian food made from cassava), bread, plain cakes, cookies (without filling), pasta, instant noodles, and cereal-based dishes (lasagna, pancakes), etc. The “vegetables and seasonings” pattern was characterized by the presence of pumpkin, gherkin, carrot, okra, tomato, cucumber, onion, bell pepper, chayote, beetroot, garlic, seasoning (cumin), paprika, salt, vinegar, industrialized tomato sauce, ketchup, pepper, industrialized seasonings, cinnamon, cloves, bay leaves, and worcestershire sauce.

The foods comprising the “sugars and fast food” pattern included sugars (brown, crystal, demerara), powdered juices already sweetened, sweets, stuffed biscuits, soft drinks, cakes with filling and/ or covering, candies, chewing gum, sweeteners, snacks, dairy drink, chocolate milk, ice cream, popsicle, condensed milk, coxinha, salted pie, pastel, croissant, pie, among others. The “alcohol and greens” pattern included the consumption of alcoholic beverages in general (beer, cachaça, wine) and vegetables (lettuce, cabbage, kale, cilantro, green onions, mint, oregano).

The “dairy and soups” pattern included the consumption of milk (whole/skim), cheeses, and fruit shakes made with milk; homemade soups, chicken soup, and meat broths. The “fruit” pattern was characterized by a higher consumption of fruits (banana, orange, apple, guava, mango, lemon, pineapple, watermelon, coconut, etc.), fresh fruit juices, and coconut water.

### Characteristics of the study participants

A total of 1,608 women were identified in the selected communities. Of these, 588 (36.6%) were excluded due to missing data for MetS classification; 61 (3.8%) due to lactation; 47 (2.9%) for having consumed alcoholic beverages on the day of the biochemical examination; and 17 (1.1%) for having implausible caloric intake. Thus, the final sample consisted of 895 women (mean age: 38.9 ± 11.0 years). The ratio of individuals to food groups used to derive dietary patterns in this study was approximately 64:1, meeting the assumptions for sample size for factor analysis.

The socioeconomic, anthropometric, and lifestyle profiles of the excluded women (*n* = 713; 44.3%) did not differ significantly from those observed in the analyzed women (*n* = 895; 55.7%). In this regard, similarities (non-significant differences according to the chi-square test; *p* > 0.05) were observed in the frequencies of the following conditions: less than 8 years of schooling, race/skin color, food insecurity, sedentary physical activity pattern, and overweight. Therefore, the possibility of bias due to significant differences between the characteristics of the analyzed and non-analyzed women is reduced.

The majority of participants in this study were Black (90.8%), belonged to economic class D + E (94.4%), had less than 8 years of education (71.0%), and 69% were overweight (Tables 3 and 4).

**Table 3:** Metabolic syndrome by demographic and socioeconomic characteristics in Quilombola women, Alagoas, Brazil, 2018

Variables^+^	Total (*n* = 895)	Metabolic syndrome		Crude PR (95% CI)	*p* ^a^
		No (*n* = 463)	Yes (*n* = 432)		
	*n* (%)	*n* (%)	*n* (%)		
Age group (years) (*n* = 895)					
19 a 29	211 (23.2)	170 (80.6)	41 (19.4)	1	1
30 a 39	263 (29.4)	144 (54.8)	119 (45.3)	2.33 (1.72–3.16)	< 0.001*
40 a 49	238 (26.6)	96 (40.3)	142 (59.7)	3.07 (2.29–4.12)	< 0.001*
50 a 59	183 (20.5)	53 (29.0)	130 (71.0)	3.66 (2.74–4.89)	< 0.001*
Race/skin color (self-reported) (*n* = 894)					
Different from brown or black	82 (9.2)	37 (45.1)	45 (54.9)	1	-
Black or Brown	812 (90.8)	425 (52.3)	387 (47.7)	0.87 (0.70–1.07)	0.186*
Marital status (*n* = 895)					
Single	111 (12.4)	72 (64.9)	39 (35.1)	1	-
Married (lives with a partner)	711 (79.4)	354 (49.8)	357 (50.2)	1.43 (1.10–1.86)	0.008*
Widowed/divorced	73 (8.2)	37 (50.7)	36 (49.3)	1.40 (0.99–1.97)	0.053*
Schooling (complete years of study) (*n* = 892)					
≥ 8	259 (29.0)	180 (69.5)	79 (30.5)	1	-
Never studied	112 (12.6)	39 (34.8)	73 (65.2)	2.14 (1.70–2.69)	< 0.001*
1–4	364 (40.8)	153(42.0)	211 (58.0)	1.90 (1.56–2.33)	< 0.001*
5–7	157 (17.6)	88 (56.1)	69 (43.9)	1.44 (1.12–1.86)	0.005*
Economic class¹ (*n* = 894)					
B + C	50 (5.6)	30 (60.0)	20 (40.0)	1	-
D + E	844 (94.3)	433 (51.3)	411(48.7)	1.22 (0.86–1.72)	0.266
Family participation in a government program (*n* = 895)					
No	236 (26.4)	97 (41.1)	139 (58.9)	1	-
Yes	659 (73.6)	366 (55.5)	293 (44.5)	0.75 (0.66–0.87)	< 0.001*
Family income (in minimum wage)^b^ (*n* = 716)					
> 1	248 (34.6)	138 (55.7)	110 (44.3)	1	-
≤ 1	468 (65.4)	232 (49.6)	236 (50.4)	1.14 (0.96–1.34)	0.130*
Employment status (*n* = 888)					
Employed	507 (57.1)	250 (49.3)	257 (50.7)	1	-
Unemployed	381 (42.9)	209 (54.9)	172 (45.1)	0.89 (0.77–1.02)	0.105*
Food and nutritional insecurity (*n* = 884)					
No	515 (58.3)	278 (54.0)	237 (46.0)	1	-
Yes	369 (41.7)	182 (49.3)	187 (50.7)	1.10 (0.96–1.26)	0.169*

^+^Denominators may vary due to missing values for specific variables; PR = prevalence ratios; 95% CI = 95% confidence intervals; MetS= metabolic syndrome

^a^ Pearson χ 2 test

^1^ According to the Criterion for Economic Classification Brazil (There were no families in class A)

^b^ Minimum wage at the time of the survey: R$937.00 (US$293.70)

^*^ Variable selected to compose the multivariate analysis (*p* < 0.2)

**Table 4:** Metabolic syndrome by lifestyle, health, and anthropometric characteristics in Quilombola women, Alagoas, Brazil, 2018

Variables^1^	Total (*n* = 895)	Metabolic syndrome		Crude PR (95% CI)	*p* ^a^
		No (*n* = 463)	Yes (*n* = 432)		
	*n* (%)	*n* (%)	*n* (%)		
Smoking (*n* = 887)					
No	684 (77.1)	380 (55.6)	304 (44.4)	1	-
Yes	203 (22.9)	81 (39.9)	122 (60.1)	1.35 (1.17–1.55)	< 0.001*
Alcoholism (*n* = 887)					
No	578 (65.2)	292 (50.5)	286 (49.5)	1	-
Yes	309 (34.8)	169 (54.7)	140 (45.3)	0.91 (0.79–1.06)	0.242
Physical activity level (*n* = 890)					
Active	531 (59.7)	272 (51.2)	259 (48.8)	1	-
Sedentary	359 (40.3)	186 (51.8)	173 (48.2)	0.99 (0.86–1.13)	0.864
Health problems in the last 15 days (*n* = 894)					
No	589 (65.9)	315 (53.5)	274 (46.5)	1	-
Yes	305 (34.1)	147 (48.2)	158 (51.8)	1.11 (0.97–1.28)	0.128*
Overweight (BMI ≥ 25 kg/m^2^) (*n* = 873)					
No	271 (31.0)	228 (84.1)	43 (15.9)	1	-
Yes	602 (69.0)	220 (36.5)	382 (63.5)	3.99 (3.02–5.30)	< 0.001*
Waist-to-height ratio (*n* = 883)					
Normal	336 (38.1)	282 (84.0)	54 (16.0)	1	-
High (≥ 0,53)	547 (61.9)	174 (37.8)	373 (68.2)	4.24 (3.30–5.45)	< 0.001*
Neck circumference (*n* = 882)					
Normal	503 (57.0)	344 (68.4)	159 (31.6)	1	-
High (≥ 34 cm)	379 (43.0)	111 (29.3)	268 (70.7)	2.24 (1.94–2.58)	< 0.001*

^1^Denominators may vary due to missing values for specific variables; PR = prevalence ratios; IC95% = 95% confidence intervals

^a^ Pearson χ^2^ test

^*^Variable selected to compose the multivariate analysis (*p* < 0.2)

In the unadjusted analysis, the covariates associated with a higher prevalence of MetS were older age, low education, married status, participation in government programs, smoking, overweight, and elevated WHtR and neck circumference (Tables 3 and 4).

MetS was identified in 48.3% of women, and regarding its components, 68.2% had abdominal obesity, 45.0% hypertension, 28.8% hyperglycemia, 74.9% low HDL, and 33.1% hypertriglyceridemia (Supplementary Table 2).

### Associations between dietary patterns and metabolic syndrome and its components

#### Crude analysis

In the crude analysis, it was observed that consumption in the fourth quartile (Q4) of the “vegetables and seasonings” pattern was associated with a lower prevalence of MetS (PR: 0.81; 95% CI: 0.67–0.98) compared to the lowest quartile (Table 5). However, this association did not remain statistically significant in the multiple analysis. Therefore, no dietary pattern was independently associated with MetS in the final model (Table 6).

**Table 5:** Crude prevalence ratios between dietary patterns and metabolic syndrome in Quilombola women, Alagoas, Brazil, 2018

Variables	Total	Metabolic syndrome (*n* = 895)		PR (CI 95%)	*p* ^a^
		No	Yes		
	*n* (%)	*n* (%)	*n* (%)		
Meat and beans pattern					
Q1	224 (25.0)	102 (50.0)	112 (50.0)	1.00	1
Q2	224 (25.0)	110 (49.1)	114 (50.9)	1.02 (0.85–1.22)	0.850
Q3	224 (25.0)	121 (54.0)	103 (46.0)	0.92 (0.76–1.12)	0.395
Q4	223 (24.9)	120 (53.8)	103 (46.2)	0.92 (0.76–1.12)	0.421
Cereals/roots, oils, and infusions pattern					
Q1	224 (25.0)	119 (53.1)	105 (46.9)	1.00	1
Q2	224 (25.0)	121 (54.0)	103 (46.0)	0.98 (0.80–1.20)	0.850
Q3	224 (25.0)	116 (51.8)	108 (48.2)	1.03 (0.85–1.25)	0.777
Q4	223 (24.9)	107 (48.0)	116 (52.0)	1.11 (0.92–1.34)	0.278
Vegetables and seasonings pattern					
Q1	224 (25.0)	105 (46.9)	119 (53.1)	1.00	1
Q2	224 (25.0)	114 (50.9)	110 (49.1)	0.92 (0.77–1.11)	0.396
Q3	224 (25.0)	117 (52.2)	107 (47.8)	0.90 (0.75–1.08)	0.258
Q4	223 (24.9)	127 (57.0)	96 (43.0)	**0.81 (0.67–0.98)**	**0.034**
Sugars and fast food pattern					
Q1	224 (25.0)	116 (51.8)	108 (48.2)	1	1
Q2	224 (25.0)	111 (49.6)	113 (50.4)	1.05 (0.87–1.26)	0.637
Q3	224 (25.0)	113 (50.4)	111 (49.6)	1.03 (0.85–1.24)	0.777
Q4	223 (24.9)	123 (55.2)	100 (44.8)	0.93 (0.76–1.14)	0.476
Alcohol and greens pattern					
Q1	224 (25.0)	117 (52.2)	107 (47.8)	1.00	1
Q2	224 (25.0)	126 (56.3)	98 (43.7)	0.92 (0.75–1.12)	0.394
Q3	224 (25.0)	103 (46.0)	121 (54.0)	1.13 (0.94–1.36)	0.187
Q4	223 (24.9)	117 (52.5)	106 (47.5)	0.99 (0.82–1.21)	0.960
Dairy and soups pattern					
Q1	224 (25.0)	113 (50.4)	111 (49.6)	1.00	1
Q2	224 (25.0)	121 (54.0)	103 (46.0)	0.93 (0.76–1.13)	0.450
Q3	224 (25.0)	112 (50.0)	112 (50.0)	1.01 (0.84–1.22)	0.925
Q4	223 (24.9)	117 (52.5)	106 (47.5)	0.96 (0.79–1.16)	0.669
Fruits pattern					
Q1	224 (25.0)	121 (54.0)	103 (46.0)	1.00	1
Q2	224 (25.0)	109 (48.7)	115 (51.3)	1.12 (0.92–1.35)	0.258
Q3	224 (25.0)	112 (50.0)	112 (50.0)	1.09 (0.90–1.32)	0.395
Q4	223 (24.9)	121 (54.3)	102 (45.7)	0.99 (0.81–1.22)	0.959

Boldface values indicate statistically significant associations (*p* < 0.05)

*PR* Prevalence ratios, *95% CI* 95% confidence intervals

Q1 = 1st quarter; Q2 = 2nd quarter; Q3 = 3rd quarter; Q4 = 4th quarter

^a^ Pearson χ^2^ test

**Table 6:** Adjusted prevalence ratios for the association of metabolic syndrome with dietary patterns in Quilombola women, Alagoas, Brazil, 2018

Dietary patterns/outcomes	Model 1					Model 2					Model 3				
	PR (CI 95%)				*P* ^a, b^	PR (CI 95%)				*P* ^a, b^	PR (CI 95%)				*P* ^a, b^
Meat and beans pattern	Q1	Q2	Q3	Q4		Q1	Q2	Q3	Q4		Q1	Q2	Q3	Q4	
Arterial hypertension	1.00	0.94 (0.77–1.16)	1.02 (0.83–1.24)	1.03 (0.84–1.27)	0.757	1.00	0.94 (0.76–1.17)	1.01 (0.83–1.25)	1.04 (0.85–1.29)	0.683	1.00	0.92 (0.75–1.13)	0.99 (0.82–1.21)	1.02 (0.83–1.25)	0.837
Hyperglycemia (HbA1C ≥ 6,5 mg/dL )	1.00	1.25 (0.93–1.67)	1.02 (0.74–1.39)	1.28 (0.95–1.73)	0.103	1.00	1.26 (0.93–1.69)	1.05 (0.76–1.44)	1.29 (0.95–1.75)	0.100	1.00	1.18 (0.88–1.58)	0.96 (0.70–1.31)	1.24 (0.91–1.67)	0.168
Abdominal obesity (waist circumference ≥ 80 cm)	1.00	1.02 (0.90–1.15)	1.03 (0.91–1.16)	1.02 (0.90–1.15)	0.776	1.00	1.02 (0.90–1.15)	1.03 (0.91–1.16)	1.02 (0.89–1.16)	0.780	1.00	0.99 (0.92–1.08)	0.98 (0.90–1.07)	1.01 (0.93–1.09)	0.858
HDL low (HDL < 50 mg/dL)	1.00	0.98 (0.89–1.08)	0.91 (0.82–1.02)	0.93 (0.84–1.04)	0.208	1.00	0.98 (0.88–1.08)	0.91 (0.82–1.01)	0.93 (0.84–1.03)	0.174	1.00	0.98 (0.87–1.02)	0.90 (0.81–1.01)	0.92 (0.82–1.02)	0.107
Hypertriglyceridemia (Triglycerides ≥175 mg/dL)	1.00	0.87 (0.67–1.14)	0.77 (0.59–1.01)	0.80 (0.60–1.06)	0.127	1.00	0.89 (0.67–1.16)	0.78 (0.59–1.02)	0.81 (0.61–1.08)	0.150	1.00	0.84 (0.64–1.10)	**0.76** **(0.58–0.99**)	0.80 (0.60–1.06)	**0.040**
Metabolic syndrome	1.00	0.99 (0.82–1.20)	0.89 (0.73–1.09)	0.99 (0.82–1.22)	0.987	1.00	0.99 (0.81–1.21)	0.89 (0.73–1.10)	0.99 (0.81–1.22)	0.981	1.00	0.96 (0.80–1.16)	0.89 (0.74–1.07)	0.99 (0.82–1.19)	0.894
Cereals/roots, oils, and infusions pattern															
Arterial hypertension	1.00	0.97 (0.80–1.17)	1.05 (0.87–1.26)	1.06 (0.87–1.28)	0.581	1.00	0.93 (0.79–1.17)	1.07 (0.88–1.28)	1.08 (0.89–1.31)	0.443	1.00	0.94 (0.7–1.13)	1.05 (0.88–1.26)	1.04 (0.86–1.26)	0.689
Hyperglycemia (HbA1C ≥ 6,5 mg/dL )	1.00	0.95 (0.71–1.27)	1.07 (0.80–1.41)	0.97 (0.71–1.30)	0.797	1.00	0.96 (0.72–1.28)	1.07 (0.80–1.41)	0.96 (0.71–1.30)	0.790	1.00	1.01 (0.76–1.36)	1.13 (0.85–1.50)	0.99 (0.73–1.35)	0.966
Abdominal obesity (waist circumference ≥ 80 cm)	1.00	0.99 (0.87–1.11)	0.99 (0.87–1.12)	1.11 (0.98–1.24)	0.092	1.00	0.98 (0.87–1.11)	0.99 (0.87–1.12)	1.11 (0.99–1.24)	0.085	1.00	1.06 (0.98–1.15)	1.05 (0.97–1.14)	**1.10** **(1.01–1.19)**	**0.029**
HDL low (HDL < 50 mg/dL)	1.00	0.92 (0.83–1.03)	0.98 (0.89–1.09)	0.96 (0.88–1.07)	0.478	1.00	0.93 (0.83–1.03)	0.98 (0.89–1.09)	0.96 (0.87–1.07)	0.484	1.00	0.93 (0.83–1.03)	0.99 (0.89–1.10)	0.96 (0.87–1.06)	0.432
Hypertriglyceridemia (Triglycerides ≥175 mg/dL)	1.00	0.96 (0.74–1.25)	1.11 (0.86–1.43)	1.22 (0.94–1.57)	0.129	1.00	0.98 (0.75–1.27)	1.13 (0.87–1.46)	1.23 (0.95–1.59)	0.123	1.00	0.95 (0.73–1.24)	1.13 (0.88–1.45)	1.19 (0.93–1.54)	0.170
Metabolic syndrome	1.00	0.86 (0.70–1.06)	0.96 (0.79–1.18)	1.07 (0.87–1.31)	0.158	1.00	0.87 (0.71–1.06)	0.96 (0.79–1.18)	1.08 (0.88–1.31)	0.447	1.00	0.87 (0.72–1.05)	0.98 (0.81–1.18)	1.05 (0.87–1.25)	0.610
Vegetables and seasonings pattern															
Arterial hypertension	1.00	1.04 (0.87–1.24)	1.07 (0.89–1.28)	0.95 (0.77–1.16)	0.628	1.00	1.04 (0.87–1.24)	1.07 (0.90–1.29)	0.93 (0.76–1.14)	0.486	1.00	1.02 (0.86–1.21)	1.06 (0.89–1.27)	0.90 (0.74–1.11)	0.326
Hyperglycemia (HbA1C ≥ 6,5 mg/dL )	1.00	1.02 (0.78–1.33)	0.87 (0.64–1.17)	1.05 (0.79–1.39)	0.739	1.00	1.02 (0.78–1.34)	0.87 (0.64–1.17)	1.05 (0.79–1.38)	0.754	1.00	1.03 (0.79–1.34)	0.86 (0.64–1.16)	0.97 (0.73–1.28)	0.818
Abdominal obesity (waist circumference ≥ 80 cm)	1.00	0.98 (0.88–1.11)	0.97 (0.86–1.09)	0.96 (0.85–1.09)	0.547	1.00	0.98 (0.87–1.11)	0.97 (0.86–1.08)	0.85 (0.85–1.09)	0.550	1.00	1.03 (0.95–1.12)	0.97 (0.89–1.05)	0.94 (0.87–1.02)	0.141
HDL low (HDL < 50 mg/dL)	1.00	0.91 (0.81–1.01)	0.96 (0.86–1.06)	0.91 (0.82–1.01)	0.079	1.00	0.91 (0.82–1.01)	0.96 (0.86–1.06**)**	0.91 (0.82–1.01)	0.081	1.00	0.91 (0.82–1.01)	0.96 (0.86–1.06)	0.91 (0.82–1.01)	0.061
Hypertriglyceridemia (Triglycerides ≥175 mg/dL)	1.00	0.96 (0.76–1.21)	0.86 (0.67–1.11)	0.85 (0.65–1.09)	0.204	1.00	0.97 (0.77–1.23)	0.87 (0.67–1.12)	0.85 (0.65–1.10)	0.210	1.00	0.97 (0.77–1.23)	0.85 (0.66–1.09)	0.82 (0.64–1.06)	0.131
Metabolic syndrome	1.00	1.02 (0.85–1.24)	1.03 (0.85–1.24)	0.98 (0.79–1.21)	0.861	1.00	1.03 (0.85–1.24)	1.03 (0.85–1.24)	0.98 (0.79–1.21)	0.865	1.00	1.01 (0.84–1.21)	0.98 (0.82–1.17)	0.92 (0.76–1.11)	0.383
Sugars and fast food pattern															
Arterial hypertension	1.00	1.05 (0.88–1.25)	1.03 (0.85–1.25)	1.02 (0.83–1.24)	0.866	1.00	1.04 (0.87–1.24)	1.03 (0.85–1.26)	1.01 (0.82–1.23)	0.938	1.00	1.04 (0.87–1.23)	0.97 (0.81–1.18)	0.98 (0.79–1.17)	0.740
Hyperglycemia (HbA1C ≥ 6,5 mg/dL )	1.00	1.23 (0.93–1.63)	1.27 (0.96–1.69)	1.05 (0.76–1.45)	0.762	1.00	1.23 (0.93–1.63)	1.28 (0.96–1.70)	1.05 (0.76–1.45)	0.756	1.00	1.27 (0.97–1.68)	1.24 (0.93–1.65)	1.02 (0.73–1.42)	0.911
Abdominal obesity (waist circumference ≥ 80 cm)	1.00	1.02 (0.90–1.15)	**1.13** **(1.01–1.27)**	1.06 (0.94–1.21)	**0.045**	1.00	1.02 (0.90–1.15)	**1.13** **(1.01–1.27)**	1.06 (0.94–1.20)	**0.047**	1.00	1.01 (0.93–1.10)	1.02 (0.94–1.10)	0.99 (0.91–1.07)	0.771
HDL low (HDL < 50 mg/dL)	1.00	1.04 (0.94–1.15)	0.97 (0.86–1.08)	0.99 (0.90–1.11)	0.980	1.00	1.04 (0.94–1.16)	0.97 (0.87–1.09)	1.01 (0.90–1.12)	0.985	1.00	1.05 (0.95–1.17)	0.97 (0.87–1.08)	1.01 (0.90–1.12)	0.937
Hypertriglyceridemia (Triglycerides ≥175 mg/dL)	1.00	1.07 (0.82–1.42)	1.08 (0.81–1.44)	1.13 (0.85–1.52)	0.400	1.00	1.07 (0.81–1.41)	1.09 (0.82–1.45)	1.13 (0.85–1.52)	0.397	1.00	1.06 (0.80–1.41)	1.02 (0.77–1.36)	1.12 (0.84–1.50)	0.443
Metabolic syndrome	1.00	1.01 (0.84–1.24)	1.05 (0.85–1.29)	1.06 (0.86–1.31)	0.569	1.00	1.02 (0.84–1.23)	1.06 (0.86–1.29)	1.06 (0.86–1.31)	0.573	1.00	1.01 (0.85–1.21)	0.97 (0.80–1.17)	1.02 (0.84–1.24)	0.837
Alcohol and greens pattern															
Arterial hypertension	1.00	0.97 (0.78–1.21)	1.06 (0.87–1.30)	0.95 (0.77–1.18)	0.648	1.00	0.97 (0.77–1.21)	1.06 (0.87–1.30)	0.95 (0.77–1.18)	0.646	1.00	1.05 (0.85–1.30)	1.13 (0.93–1.38)	0.99 (0.82–1.22)	0.995
Hyperglycemia (HbA1C ≥ 6,5 mg/dL )	1.00	0.87 (0.64–1.17)	1.09 (0.83–1.45)	0.92 (0.68–1.24)	0.594	1.00	0.85 (0.63–1.15)	1.09 (0.83–1.44)	0.91 (0.67–1.23)	0.521	1.00	0.91 (0.67–1.22)	1.13 (0.86–1.49)	0.88 (0.65–1.19)	0.407
Abdominal obesity (waist circumference ≥ 80 cm)	1.00	0.95 (0.84–1.07)	0.98 (0.87–1.09)	0.95 (0.84–1.07)	0.368	1.00	0.84 (0.84–1.07)	0.98 (0.87–1.09)	0.95 (0.84–1.07)	0.368	1.00	1.02 (0.94–1.10)	1.05 (0.97–1.13)	0.98 (0.90–1.06)	0.583
HDL low (HDL < 50 mg/dL)	1.00	1.08 (0.97–1.20)	0.97 (0.86–1.08)	1.05 (0.95–1.17)	0.349	1.00	1.08 (0.97–1.20)	0.97 (0.86–1.09)	1.05 (0.94–1.17)	0.348	1.00	1.10 (0.99–1.22)	0.97 (0.86–1.09)	1.07 (0.96–1.19)	0.197
Hypertriglyceridemia (Triglycerides ≥175 mg/dL)	1.00	1.19 (0.91–1.54)	1.08 (0.83–1.40)	1.12 (0.86–1.46)	0.382	1.00	1.19 (0.91–1.54)	1.06 (0.82–1.38)	1.11 (0.85–1.44)	0.446	1.00	1.24 (0.96–1.60)	1.11 (0.83–1.44)	1.19 (0.92–1.53)	0.193
Metabolic syndrome	1.00	0.98 (0.79–1.21)	1.07 (0.88–1.30)	1.06 (0.86–1.29)	0.591	1.00	0.98 (0.79–1.21)	1.08 (0.89–1.31)	1.06 (0.86–1.29)	0.594	1.00	1.07 (0.88–1.30)	1.14 (0.95–1.37)	1.07 (0.89–1.28)	0.470
Dairy and soups pattern															
Arterial hypertension	1.00	1.04 (0.85–1.29)	1.08 (0.87–1.33)	1.05 (0.85–1.31)	0.647	1.00	1.04 (0.85–1.29)	1.07 (0.86–1.32)	1.05 (0.85–1.31)	0.648	1.00	1.12 (0.91–1.36)	1.18 (0.95–1.43)	1.08 (0.87–1.33)	0.485
Hyperglycemia (HbA1C ≥ 6,5 mg/dL )	1.00	**0.65** **(0.47–0.89)**	0.98 (0.75–1.29)	1.09 (0.84–1.43)	**0.008**	1.00	**0.65** **(0.47–0.89)**	0.99 (0.75–1.29)	1.10 (0.84–1.43)	**0.007**	1.00	**0.68** **(0.49–0.93)**	1.03 (0.78–1.35)	1.12 (0.86–1.46)	**0.018**
Abdominal obesity (waist circumference ≥ 80 cm)	1.00	0.93 (0.82–1.05)	0.98 (0.88–1.10)	1.02 (0.91–1.15)	0.688	1.00	0.93 (0.82–1.05)	0.98 (0.88–1.10)	1.02 (0.91–1.15)	0.683	1.00	0.97 (0.89–1.05)	1.02 (0.94–1.10)	1.02 (0.94–1.10)	0.608
HDL low (HDL < 50 mg/dL)	1.00	**0.88** **(0.79–0.98)**	0.97 (0.88–1.07)	0.90 (0.81–1.01)	**0.021**	1.00	**0.88** **(0.79–0.98)**	0.98 (0.89–1.08)	0.90 (0.81–1.01)	**0.022**	1.00	**0.89** **(0.80–0.99)**	1.01 (0.91–1.10)	0.91 (0.82–1.01)	**0.046**
Hypertriglyceridemia (Triglycerides ≥175 mg/dL)	1.00	1.12 (0.85–1.48)	0.97 (0.71–1.32)	1.15 (0.87–1.52)	0.331	1.00	1.12 (0.85–1.48)	0.98 (0.72–1.33)	1.15 (0.87–1.52)	0.322	1.00	1.16 (0.87–1.53)	1.01 (0.74–1.37)	1.16 (0.88–1.52)	0.309
Metabolic syndrome	1.00	0.92 (0.75–1.11)	0.95 (0.78–1.16)	0.97 (0.80–1.19)	0.789	1.00	0.92 (0.75–1.12)	0.96 (0.79–1.17)	0.97 (0.80–1.18)	0.792	1.00	0.97 (0.81–1.18)	1.05 (0.88–1.26)	0.99 (0.83–1.19)	0.915
Fruits pattern															
Arterial hypertension	1.00	1.01 (0.83–1.20)	1.05 (0.87–1.26)	0.85 (0.69–1.04)	0.114	1.00	0.99 (0.83–1.20)	1.04 (0.87–1.25)	0.85 (0.69–1.04)	0.117	1.00	1.08 (0.89–1.30)	1.08 (0.90–1.29)	0.89 (0.73–1.09)	0.259
Hyperglycemia (HbA1C ≥ 6,5 mg/dL )	1.00	1.09 (0.82–1.45)	0.93 (0.69–1.26)	1.06 (0.78–1.42)	0.707	1.00	1.09 (0.82–1.45)	0.94 (0.70–1.27)	1.06 (0.80–1.43)	0.688	1.00	1.16 (0.88–1.54)	0.95 (0.70–1.28)	1.07 (0.80–1.44)	0.635
Abdominal obesity (waist circumference ≥ 80 cm)	1.00	0.97 (0.86–1.09)	0.97 (0.87–1.09)	0.93 (0.82–1.05)	0.240	1.00	0.97 (0.86–1.10)	0.97 (0.87–1.09)	0.93 (0.82–1.05)	0.221	1.00	**1.10** **(1.02–1.20)**	1.01 (0.93–1.08)	0.99 (0.92–1.07)	**0.017**
HDL low (HDL < 50 mg/dL)	1.00	0.98 (0.88–1.09)	0.92 (0.82–1.03)	1.01 (0.91–1.12)	0.825	1.00	0.98 (0.88–1.09)	0.92 (0.82–1.02)	1.02 (0.92–1.13)	0.725	1.00	0.99 (0.90–1.11)	0.92 (0.82–1.03)	1.02 (0.92–1.13)	0.724
Hypertriglyceridemia (Triglycerides ≥175 mg/dL)	1.00	1.09 (0.82–1.46)	1.05 (0.78–1.40)	1.02 (0.76–1.38)	0.877	1.00	1.08 (0.82–1.44)	1.04 (0.77–1.39)	1.02 (0.76–1.37)	0.904	1.00	1.15 (0.86–1.54)	1.07 (0.80–1.42)	1.10 (0.83–1.47)	0.505
Metabolic syndrome	1.00	1.04 (0.85–1.29)	1.05 (0.86–1.30)	1.03 (0.83–1.27)	0.794	1.00	1.05 (0.85–1.29)	1.06 (0.86–1.31)	1.03 (0.83–1.27)	0.814	1.00	1.17 (0.96–1.42)	1.07 (0.89–1.30)	1.12 (0.93–1.34)	0.242

*PR* Prevalence ratio, *95%CI* 95% confidence intervals

Q1 = 1st quarter; Q2 = 2nd quarter; Q3 = 3rd quarter; Q4 = 4th quarter

Model 1: adjusted for demographic and socioeconomic characteristics; Model 2: adjusted for variables from model 1 with *p* < 0.05 added lifestyle and health data; Model 3: adjusted for variables from models 1 and 2 with *p* < 0.05 plus anthropometric characteristics

^a^ Values reported in bold only refer to the quartile that showed association

^b^ values without bold refer to Q4 of each model

The crude analysis of MetS components (data not shown in tables) showed that consumption of the ‘meat and beans’ and ‘vegetables and seasonings’ patterns in Q4 was associated with a 26% lower prevalence of high TG (PR = 0.74; 95% CI: 0.57–0.96). Consumption in Q4 of the ‘vegetables and seasonings’ pattern was associated with lower prevalences of abdominal obesity (PR = 0.87; 95% CI: 0.77–0.99) and hypertriglyceridemia (PR = 0.72; 95% CI: 0.55–0.94) compared to Q1. Lower prevalence of hyperglycemia was associated with consumption in the second quartile (Q2) of foods from the ‘dairy and soups’ pattern (PR = 0.70; 95% CI: 0.50–0.97). Consumption in Q2 of the ‘fruits’ pattern was associated with a higher prevalence of high TG (PR: 1.35; 95% CI: 1.03–1.75) compared to Q1.

#### Multivariable analysis

In the adjusted analysis, associations were observed between some components of MetS and dietary patterns (Table 6). Moderate consumption of foods from the ‘meat and beans’ pattern (Q3 compared to Q1) was associated with a lower prevalence of high triglycerides (PR = 0.76; 95% CI: 0.58–0.99). Consumption of the ‘cereals/roots, oils, and infusions’ pattern in Q4 compared to Q1 was related to a higher prevalence of abdominal obesity (PR = 1.10; 95% CI: 1.01–1.19). Adherence to the “sugars and fast food” pattern (Q3 compared with Q1) was associated with a higher prevalence of abdominal obesity in Model 2; ; however, this association did not remain statistically significant in the final adjusted model. Consumption in the second quartile (Q2) of the ‘dairy and soups’ pattern was associated with lower prevalences of hyperglycemia (PR = 0.68; 95% CI: 0.49–0.93) and low HDL (PR = 0.89; 95% CI: 0.80–0.99) compared to Q1. It was observed that adherence in the second quartile (Q2) of the ‘fruits’ pattern was associated with a higher prevalence of abdominal obesity (PR = 1.10; 95% CI: 1.02–1.20).

## Discussion

In this study, none of the identified dietary patterns were associated with MetS after adjustment. Therefore, the absence of association with MetS should be interpreted cautiously, considering the methodological limitations inherent to the study design and dietary assessment. However, most of the dietary patterns showed associations with some of the components of MetS. Although some associations were statistically significant, the magnitude of the observed effects was modest and should therefore be interpreted with caution. Women with moderate adherence to the ‘meat and beans’ pattern had a lower prevalence of hypertriglyceridemia than those with lower adherence. Higher adherence to the ‘cereals/roots, oils, and infusions’ pattern was associated with a higher prevalence of abdominal obesity. Adherence in Q2 to the ‘dairy and soups’ pattern was associated with a lower prevalence of hyperglycemia and low HDL cholesterol. Additionally, women with adherence in the second quartile (Q2) to the ‘fruits’ pattern exhibited a higher prevalence of abdominal obesity.

The dietary pattern “meat and beans” included various types of beans, eggs, and various meats. Therefore, this pattern is characterized by a high protein content (both plant-based and animal) but also by the presence of saturated fat, which is not considered healthy. In other studies, this pattern has been referred to as the “Southern” pattern [48] or “Western” [9, 11, 15].

There are reports in the literature suggesting that the consumption of saturated fat found in animal-based foods in this pattern may be associated with an increased risk of developing dyslipidemia and high blood pressure [49].

Controversially, the results of this study indicate *that moderate adherence to this dietary pattern was associated with a lower prevalence of hypertriglyceridemia* (a condition often associated with excessive carbohydrate intake), without a significant impact on overall lipoprotein levels. Data from a meta-analysis revealed that consuming more than three servings of red meat per day did not have a notable effect on hypertriglyceridemia, blood pressure, and lipoproteins, except for higher HDL concentrations [50]. Furthermore, the inverse association observed between this pattern and hypertriglyceridemia in the present study may be partly explained by the presence of the beans group in this dietary pattern, whose regular consumption is widely recognized as a healthy dietary practice [51]. In this context, the role of dietary factors in lipid metabolism remains incompletely understood [52]. However, concerning hypertriglyceridemia, diet may play an important role in triglyceride metabolism. Therefore, dietary management may represent a potential strategy to help prevent this outcome [53].

Beans are recognized as an important source of phytochemicals with hypolipidemic activity. Their components include dietary fiber, oligosaccharides, resistant starch, and phenolic compounds, which may contribute to reducing in triglyceride levels [54]. A systematic review and meta-analysis of randomized clinical trials has highlighted the health benefits of bean consumption. It has been observed that incorporating beans in the diet may be associated with reductions in LDL cholesterol levels, decreased risk of cardiovascular diseases (CVD), and lower incidence of coronary heart disease [55]. The evidence presented here suggests that the relationship between similar dietary patterns may vary among studies, which may be related to differences in ethnicity, gender, dietary culture, and lifestyle, among other factors, across populations.

Foods such as fruits, vegetables, legumes, cereals, tubers, and roots are commonly included in dietary patterns associated with healthier eating behaviors and have been widely investigated in relation to cardiometabolic health outcomes. However, in dietary pattern analysis, these foods should be interpreted within the broader combinations in which they are consumed rather than as isolated components of the diet. In the present study, the dietary pattern “cereals/roots, oils, and infusions”, despite including foods traditionally considered part of a healthy diet, such as cereals, roots, and tubers, also comprised items with different levels of processing, including products such as instant noodles. This combination reflects the nature of dietary patterns derived from principal component analysis, which identify foods that tend to be consumed together within the population rather than categorizing foods according to their individual nutritional quality [16, 45]. Thus, the identified pattern may represent a mixed dietary behavior reflecting the current food environment of quilombola communities, where traditional foods coexist with processed and ultra-processed products as part of an ongoing dietary transition observed in several traditional populations [56]. Furthermore, this dietary pattern was associated with a higher prevalence of abdominal obesity in the present study.

Additionally, oils and fats were present in this pattern. While excessive intake of fats may contribute to high dietary energy density, it is important to recognize that oils and fats also play a role in traditional culinary practices in quilombola communities, where foods are commonly prepared using local ingredients and traditional cooking methods, such as frying in oil [57]. Therefore, their presence within this pattern should not be interpreted solely as an indicator of unhealthy eating behavior but rather as part of the broader dietary context of this population. In this sense, the coexistence of traditional culinary practices with the increasing availability of processed and ultra-processed foods may contribute to hybrid dietary patterns that combine elements of traditional and contemporary food environments.

This dietary pattern may therefore be interpreted as a relatively energy-dense dietary combination, and its components should be interpreted with caution, since diets with high energy density have been associated with adipose tissue accumulation, particularly in the abdominal region [58, 59].

It was observed that low fruit consumption was associated with a higher prevalence of abdominal obesity in women. This result is consistent with a study conducted with women in southern Brazil, where low consumption of the fruit dietary pattern was identified as a risk factor for overall obesity [60]. Another study conducted with obese individuals indicated that increasing fruit intake was associated with reductions in waist circumference in women [61]. These findings align with recommendations to increase fruit consumption due to their nutritional properties (rich in fiber, antioxidants, and micronutrients), which characterize them as healthy foods [62].

However, the results observed here for the fruits pattern may have been influenced by the presence of residual confounding factors, such as hormonal changes to which women over 35 years of age may be susceptible, as well as several other determinants of obesity that were not evaluated in this study, and therefore were not controlled in the analysis [60].

It is noteworthy that this pattern consists of various fruits, natural fruit juices, and coconut water. However, specifically regarding fruit juice consumption, the results in the literature are mixed. A study with postmenopausal women showed that a long-term increase in the consumption of 100% natural fruit juice was associated with weight gain [63]. An investigation in Eastern Europe assessing fruit juice consumption found that participants in Russia who drank fruit juice every day had a higher BMI compared with those who did not adopt this practice, while the opposite result (lower BMI) was found among Czech participants who consumed fruit juice daily [64]. Therefore, further studies are needed to provide additional evidence regarding the relationship between this dietary pattern and overall obesity, particularly abdominal obesity, which is an important component of MetS.

Furthermore, our findings highlight the intricate relationships between dietary patterns and obesity, particularly when considering multivariable analyses adjusted for several potential confounding factors [60].

Despite being rich in vitamins and minerals, it is worth noting that the calorie content of fresh fruit juices is similar to that of sugary beverages, although they differ in that they contain micronutrients and phytonutrients that are not present in these drinks [65, 66]. Another issue is that fruit juices are rich in fructose, whose consumption has been associated with alterations in blood lipid profiles, inflammation markers, and blood pressure [67]. Additionally, fructose intake may stimulate hormonal responses that favor visceral fat deposition and, consequently, abdominal obesity [67, 68].

The sugar and fast food pattern resembles Western patterns observed in other studies, characterized by foods rich in fat, cholesterol, and refined carbohydrates [9, 15, 69, 70]. Additionally, because it consists of ready-to-eat foods such as pizza, snacks, soft drinks, packaged juices, filled and frosted cakes, and pies, among others, it is also known as the convenience food pattern [48, 71].

Results from other studies indicate that this type of Western/fast food dietary pattern has been associated with a higher risk of central obesity [72, 73]. Furthermore, a study involving an Afro-descendant population observed that high adherence to the “fast food” pattern is a risk factor for hypertension, diabetes, MetS, and low HDL-C [74].

In the present study, this pattern lost its positive association with abdominal obesity in the final adjusted model. However, it is important to note that abdominal adiposity is associated with elevated blood pressure, diabetes mellitus, and dyslipidemias, which are factors related to cardiovascular disease risk and metabolic complications [75]. A study with Korean adults reported a higher prevalence of abdominal obesity among individuals with a higher consumption of the pattern called “meats and fast food” [72]. In contrast, Fröhlich et al. [59]., in a study in southern Brazil, observed that women with abdominal obesity had lower adherence to the “snacks/fast-food” pattern. This could be explained by possible changes in the dietary habits among the study population due to increased awareness of abdominal obesity through public health initiatives and strategies.

Therefore, even though there is a strong relationship between abdominal adiposity, unhealthy eating habits, and the development of NCDs, more studies with diverse populations are needed to better understand these associations.

Furthermore, it is worth highlighting that an important aspect to be analyzed is the level of physical activity of individuals belonging to “sugar and fast food” eating patterns, as physically active individuals tend to have a lower prevalence of obesity [76, 77]. In the case of this study, the majority of women (almost 60%) were physically active, and concerning consumption of the aforementioned dietary pattern, most fall into the moderate to high consumption category (these data were not presented in tables or mentioned in the results).

Adherence to the “dairy and soups” pattern was associated with a lower prevalence of hyperglycemia and low HDL. Randomized clinical trials have shown that dairy and dairy products, due to their calcium content, can help reduce central obesity and insulin resistance [78, 79].

A study conducted with adults in Brazil observed that for women, a higher consumption of dairy products, especially cheese, was associated with higher HDL levels [80]. Despite these findings, the relationship between dairy products and plasma lipids still warrants further investigation.

The lack of association between dietary patterns and MetS was also found in a study with adults from Poland [15].

However, a systematic review revealed that the “Meat/Western” pattern was associated with an increased risk of MetS by 20% in Asia, 15% in Europe, and 33% in America [11].

Possibly, these contradictory results are related to the fact that MetS is a heterogeneous, complex disorder whose pathophysiology is not yet fully understood. Additionally, dietary patterns vary according to age, ethnicity [81], culture, and other lifestyle-related factors [15].

It is important to note that the patterns identified here combined both healthy and less healthy foods; therefore, no dietary pattern was entirely health-promoting or entirely composed of foods associated with a higher disease risk. This characteristic reflects the nature of dietary patterns derived from factor analysis using principal component analysis, which groups foods and beverages according to their correlation structure and how they are commonly consumed together within the population, rather than as isolated dietary exposures [82, 83]. Thus, alcoholic beverages were retained as part of the dietary patterns, reflecting their correlation with other foods and beverages in the dietary habits of the studied population. The interaction between components may weaken the associations with the dependent variables, which partially explains the lack of statistical significance for some expected relationships. In this context, the interpretation of dietary patterns should also consider the degree of food processing and the broader food environment, as emphasized in the “Guia Alimentar para a População Brasileira” (Dietary Guidelines for the Brazilian Population) [84], which highlights the importance of promoting diets based predominantly on in natura or minimally processed foods while limiting the consumption of ultra-processed products. Strengthening the dissemination and adoption of these guidelines within food and nutrition policies may contribute to improving dietary quality while respecting the cultural and culinary traditions of quilombola communities [59, 85].

It is worth highlighting that the dietary patterns identified in this study were derived from an exploratory a posteriori approach using food groups based on the 2006 Dietary Guidelines for the Brazilian Population (Brazil, 2006) [37]. Because this guide organizes foods by culinary use and nutritional function, and not by level of processing or preparation type, some food groups naturally include items with diverse nutritional profiles. This characteristic is inherent to the grouping structure adopted and reflects the real dietary context of quilombola women. The quilombola communities were formed throughout history by individuals who, deprived of their most basic rights, sought not only literal freedom but also the preservation of their culture and general way of life. Originally, they maintained a close relationship with subsistence agriculture, hunting, fishing, and the raising of small animals. Through these activities, they primarily obtained their food from natural sources, which was favorable for their food security and nutrition [6, 86].

Over the years, the dietary habits of this population have been changing due to environmental degradation, agricultural conflicts leading to reduced access to land, and the high cost of maintaining agricultural practices (such as purchasing tools and crop losses due to drought, among others) [6, 87].

A key point in this process of dietary transition is related to globalization and urbanization of small towns and rural areas (where quilombola communities are located), leading to easier access to ultra-processed foods [6]. Thus, this population combines social vulnerability [6] with exposure to dietary factors associated with the development of MetS and its components. These aspects highlight the need for public health actions aimed at promoting health and encouraging healthy eating, taking into account the culture of these individuals.

The main strength of this study is that it was the first study conducted to analyze dietary patterns in a representative sample of quilombola communities in a Brazilian state. Furthermore, it employed a posteriori dietary patterns, which are widely used in nutritional epidemiology and provide results that are more consistent with real dietary behaviors compared to patterns determined *a* priori [15, 59]. Thus, the results obtained here can serve as a parameter for understanding processes that occur in scenarios composed of similar populations.

Because this is a cross-sectional study, the observed associations should not be interpreted as causal relationships. As a limitation of this design, dietary intake was assessed using a single 24hDR, which does not allow the estimation of usual intake because it fails to capture intra-individual variability in food consumption. Consequently, no statistical adjustment for intra-individual variability could be performed. Therefore, the dietary information obtained reflects short-term dietary intake rather than habitual dietary patterns. This limitation may introduce random measurement error and potential non-differential misclassification of dietary exposure, which tends to attenuate the magnitude of associations between dietary patterns and metabolic outcomes and may partly explain the absence of statistically significant associations between dietary patterns and MetS in the present study. Nevertheless, when the objective is to describe dietary patterns at the population level, the use of a single 24hDR is considered acceptable in cross-sectional studies, particularly in large epidemiological surveys. Furthermore, similar methodological approaches have been adopted in other cross-sectional investigations assessing dietary patterns in population-based samples [88–90].

Additionally, the substitution of fasting plasma glucose with HbA1c in the definition of metabolic syndrome may limit direct comparability with studies that strictly apply the original JIS criteria. Furthermore, the use of HbA1c ≥ 6.5% identifies diabetes rather than milder forms of dysglycemia, which may underestimate less severe alterations in glucose metabolism. Finally, the use of stepwise regression to select covariates may have introduced some degree of model instability or overfitting, which should therefore be considered when interpreting the estimated associations.

Moreover, multiple statistical comparisons were performed across several dietary patterns and metabolic outcomes, which may increase the probability of type I error. In addition, sensitivity analyses using alternative factor retention strategies and rotation methods suggested a solution with fewer dietary patterns. However, these solutions resulted in dietary patterns with lower nutritional and cultural interpretability, particularly due to the merging of food groups that represented distinct dietary behaviors in quilombola women. Therefore, the seven-factor solution was retained because it better captured the diversity of dietary practices observed in the studied population, although some later components may exhibit lower stability.

## Conclusion

Seven dietary patterns were identified among the quilombola women (meat and beans pattern; cereals/roots, oils, and infusions pattern; vegetables and seasonings pattern; sugars and fast food pattern; alcohol and greens pattern; dairy and soups pattern; and fruits pattern), none of which were associated with MetS. However, some dietary patterns were associated with specific components of MetS. The “dairy and soups” pattern was associated with a lower prevalence of hyperglycemia and low HDL cholesterol. Higher adherence to the “meat and beans” pattern was associated with a lower prevalence of hypertriglyceridemia. In contrast, higher adherence to the “cereals/roots, oils, and infusions” pattern and adherence to the second quartile of the “fruits” pattern were associated with a higher prevalence of abdominal obesity.

These findings suggest the importance of strengthening public health actions aimed at promoting healthy eating and improving access to dietary guidelines for the population, particularly among socially vulnerable groups such as quilombola communities.

## Supplementary Information


Supplementary Material 1


## Data Availability

The datasets used and/or analysed during the current study are available from the corresponding author upon reasonable request.

## Abbreviations

95% CI: 95% confidence intervals
BMI: Body Mass Index
CCEB: Criterion for Economic Classification Brazil
EBIA: Brazilian Scale of Food Insecurity
HbA1C: Glycated hemoglobin
HDI: Human Development Index
HDL: High-density lipoprotein cholesterol
IPAQ: International Physical Activity Questionnaire
JIS: Joint Interim Statement
MetS: Metabolic syndrome
NC: Neck circumference
NCDs: Chronic non-communicable diseases
OR: Odds ratio
PAL: Physical activity level
POF: Household Budget Survey
PR: Prevalence ratio
PCA: Principal component analysis
KMO: Kaiser-Meyer-Olkin
Q1: 1st quarter
Q2: 2nd quarter
Q3: 3rd quarter
Q4: 4th quarter
24hDR: 24-hour dietary recall
TG: Triglycerides
WC: Waist circumference
WHtR: Waist-to-height ratio
