# Supplementary material for: Association between dietary patterns and metabolic syndrome and its components in quilombola women: A population-based cross-sectional study in Alagoas, Northeast Brazil
Source: BMC Nutr. 2026 Jun 19;12:180. doi: 10.1186/s40795-026-01402-4 (PMC13531850; doi:10.1186/s40795-026-01402-4)
Supplement: Supplementary file 1 [file 40795_2026_1402_MOESM1_ESM.docx]

**SUPPLEMENTAL FILE**

**Supplementary Table 1-** Sensitivity analysis using a four-factor solution obtained by parallel analysis among quilombola women

| **Food groups*** | **Factor 1** | **Factor 2** | **Factor 3** | **Factor 4** |
| --- | --- | --- | --- | --- |
| Sugars and candies |  |  | 0.738 |  |
| Coffee/infusions |  | 0.703 |  |  |
| Meats and eggs | 0.733 |  |  |  |
| Cereals, breads, pasta, roots, tubers and derivatives | 0.448 | 0.572 |  |  |
| Condiments/ seasonings and sauces | 0.503 |  | 0.421 |  |
| Vegetables and greens |  |  |  | 0.692 |
| Legumes/beans | 0.593 |  |  |  |
| Oils and fats |  | 0.684 |  |  |
| Natural juices |  |  |  | 0.749 |
| Fast food and snacks |  |  | 0.662 |  |
| **Eigenvalue** | **1.688** | **1.273** | **1.220** | **1.035** |
| **Variance explained (%)** | **16.9** | **12.7** | **12.2** | **10.4** |

**Footnote:** Sensitivity analyses were performed using principal component factor analysis with Varimax and Promax rotations. The table presents the four-factor solution obtained with Varimax rotation, while similar factor structures were observed using Promax rotation. Parallel analysis (Horn’s method) suggested retention of four factors. **Total variance explained:** 52.2% ***** Some food groups were reorganized or excluded in the sensitivity analyses to obtain a moderate factor structure.

**Supplementary Table 2 –** Description of MS components in quilombola women from Alagoas, Brazil, 2018.

| **Variables^*^** | | **Total (n=895)**  **n (%)** | **Metabolic syndrome** | |
| --- | --- | --- | --- | --- |
|  |  |  | **No (n=463)**  **n (%)** | **Yes (n=432)**  **n (%)** |
| **Abdominal obesity (n= 883)** | |  |  |  |
|  | No | 281 (31.8) | 264 (94.0) | 17 (6.0) |
|  | Yes | 602 (68.2) | 192 (31.9) | 410 (68.1) |
| **Arterial hypertension (n= 886)** | |  |  |  |
|  | No | 487 (54.9) | 369 (75.8) | 118 (24.2) |
|  | Yes | 399 (45.0) | 90 (22.6) | 309 (77.4) |
| **Hyperglycemia (n= 858)** | |  |  |  |
|  | No | 611 (71.2) | 416 (68.1) | 195 (31.9) |
|  | Yes | 247 (28.8) | 33 (13.4) | 214 (86.6) |
| **Hypertriglyceridemia (n= 883)** | |  |  |  |
|  | No | 591 (66.9) | 436 (73.8) | 155 (26.2) |
|  | Yes | 292 (33.1) | 27 (9.3) | 265 (90.7) |
| **HDL low (n= 881)** | |  |  |  |
|  | No | 221 (25.1) | 191 (86.4) | 30 (13.6) |
|  | Yes | 660 (74.9) | 272 (41.2) | 388 (58.8) |

* Denominators may vary due to missing values for specific variables.
